# Effects of Methylation Status of CpG Sites within the HPV16 Long Control Region on HPV16-Positive Head and Neck Cancer Cells

**DOI:** 10.1371/journal.pone.0141245

**Published:** 2015-10-28

**Authors:** Chunlin Zhang, Zeyi Deng, Xiaoli Pan, Takayuki Uehara, Mikio Suzuki, Minqiang Xie

**Affiliations:** 1 Department of Otorhinolaryngology, Head and Neck Surgery, Zhujiang Hospital, Southern Medical University, Guangzhou, China; 2 Department of Otorhinolaryngology, Head and Neck Surgery, University of the Ryukyus, Okinawa, Japan; 3 Department of Otorhinolaryngology, Head and Neck Surgery, Affiliated hospital of Zunyi Medical University, Zunyi, China; 4 Department of Otorhinolaryngology, Head and Neck Surgery, The First People’s Hospital of Guangzhou, Guangzhou, China; University of Cincinnati College of Medicine, UNITED STATES

## Abstract

**Objective:**

To map comprehensively the methylation status of the CpG sites within the HPV16 long control region (LCR) in HPV-positive cancer cells, and to explore further the effects of methylation status of HPV16 LCR on cell bioactivity and E6 and E7 expression. In addition, to analyze the methylation status of the LCR in HPV-positive oropharyngeal squamous cell carcinoma (OPSCC) patients.

**Methods and Materials:**

Methylation patterns of HPV16 LCR in UM-SCC47, CaSki, and SiHa cells and HPV16-positiive OPSCC specimens were detected by bisulfite-sequencing PCR and TA cloning. For cells treated with 5-aza-2′-deoxycytidine and E6 and E7 knockdown, MTS and trypan blue staining, annexin-V and 7-AAD staining, and prodidium iodide were used to evaluate cell growth and cell proliferation, cell apoptosis, and cell cycle arrest, respectively. E6 and E7 mRNA and protein expression were analyzed by quantitative real-time PCR and immunocytochemistry, respectively.

**Results:**

Hypermethylation status of the LCR in UM-SCC47 (79.8%) and CaSki cells (90.0%) and unmethylation status of the LCR in SiHa cells (0%) were observed. Upon demethylation, the cells with different methylation levels responded differently during growth, apoptosis, and cell cycle arrest, as well as in terms of their E6 and E7 expression. In HPV16-positive OPSCC patients, the methylation rates were 9.5% in the entire LCR region, 13.9% in the 5′-LCR, 6.0% in the E6 enhancer, and 9.5% in the p97 promoter, and hypermethylation of p97 promoter was found in a subset of cases (20.0%, 2/10).

**Conclusions:**

Our study revealed two different methylation levels of the LCR in HPV16-positive cancer cells and OPSCC patients, which may represent different carcinogenesis mechanisms of HPV-positive cancers cells. Demethylating the meCpGs in HPV16 LCR might be a potential target for a subgroup of HPV16-positive patients with head and neck squamous cell carcinoma.

## Introduction

Persistent infection with high-risk human papillomavirus (HPV) has been established as an etiologic factor in addition to excessive tobacco and alcohol consumption for head and neck squamous cell carcinoma (HNSCC) [1–4]. This applies to oropharyngeal squamous cell carcinoma (OPSCC) in particular; 50–70% of OPSCC patients are infected with HPV16 [2–7]. E6 and E7 are the two main viral oncoproteins responsible for the maintenance of HPV-mediated malignant transformation through their interactions with several important cellular proteins, such as p53 and pRb [8,9]. E2 protein can contribute to multiple biological processes including viral transcription and viral DNA replication [10–13], and induce growth arrest and cell apoptosis via its effects on the expression of E6 and E7 and other viral proteins [14–16]. All these activities of E2 are dependent on its ability to bind to the viral DNA genome, especially the early promoter p97 at specific E2-binding sites (E2BSs) located within the long control region (LCR) of the HPV genome [15,17]. The enhancer, located at the 5′-end of the p97 promoter, also contributes to the regulation of E6 and E7 expression [12].

Previous studies have demonstrated the integration of viral genomes into the host genome is often associated with disruption of the E2 gene, leading to uncontrolled expression of the E6 and E7 oncoproteins [15,18,19], Wilson et al found significant enrichment of potential integration sites within the E2 region, suggesting that E2 was also a common location of disruption upon integration into the host genome in HNSCC [19]. However, a series of studies showed that many malignant HPV-associated carcinomas lack integrated viral genome copies or include integrated viral genomes accompanied by episomal viral genomes. Even if some viral genomes are fully integrated, the E2 gene may be intact and multiple copies of the HPV genome are retained in tandem arrays, also called “concatemers,” such as the HPV16-infected CaSki cell line [20]. Thus, attempts have been made to understand other mechanisms, including methylation or sequence variations in the LCR, that regulate E6 and E7 expression [7,21,22].

DNA methylation refers to the transfer of a methyl group to cytosine residues that usually leads to gene silencing by either physically inhibiting the binding of transcription factors or chromatin structure remodeling [15,23]. The methylation status of the HPV16 genome, which contains 15 dinucleotide CpG sites in the LCR, in cervical cancer cell lines and clinical samples has been analyzed, but the roles of HPV genome methylation in carcinogenesis remain unclear [24–29]. Increased methylation of the HPV genome has been found consistently at most sites in cervical carcinoma or high-grade cervical intraepithelial neoplasia (CIN) compared with low-grade CIN [24,25]. Methylation of the HPV genome has emerged as a biomarker for cancer progression in cervical cancer [15,24,30,31]. Furthermore, some researchers reported that the hypomethylation status of CaSki cells induced by a DNA methylation inhibitor reduced cellular viability but did not affect bioactivity in HeLa cells containing a completely unmethylated HPV-18 genome [27], which inspired us to explore the potential novel effects of HPV methylation status in HPV-positive cancers.

To date, however, there is no information about the effects of methylation variation and its regulation mechanism in HNSCC cells. Moreover, since methylated CpG (meCpG) is potentially reversible, demethylating the meCpGs of the HPV genome may be a valuable target for cancer therapy. To fill this gap in knowledge, we utilized HNSCC cells for the first time to map comprehensively the methylation status of the CpG sites within the LCR, and to explore further the effects of demethylating HPV16 LCR on cell bioactivity and E6 and E7 expression. In addition, very few studies examined HNSCC specimens and their results are inconsistent [19,32,33]; we therefore showed the methylation status of the LCR in HPV-positive OPSCC patients.

## Materials and Methods

### Cell culture and 5-aza-2′-deoxycytidine (5-aza-dc) treatment

The HNSCC cell line UM-SCC47, which was developed from a patient with lateral tongue cancer [34] (a gift from Professor Thomas E. Carey, University of Michigan), and the cervical cancer cell lines CaSki (ECACC, Salisbury, UK) and SiHa (ATCC, Tokyo, Japan) were characterized in this study (Table 1). An integrated HPV16 genome was detected in the UM-SCC47, CaSki, and SiHa cell lines, which contain 15, 600, and 1–2 copies of the HPV16 genome, respectively [35,36]. The cells were cultured in RPMI1640 (for UM-SCC47 and CaSki) or EMEM (for SiHa) containing 10% fetal bovine serum and 100 IU/mL penicillin/streptomycin. The demethylation reagent 5-aza-dc (Sigma, St. Louis, MO) was dissolved in DMSO at 50 mM and stored in aliquots at −80°C until use. Exponentially growing cells were treated with 5-aza-dc at different concentrations (0.1–5 μM) for 72–120 h. The culture medium containing freshly prepared 5-aza-dc was replaced every 24 h.

**Table 1:** Origin and pathological characteristics of HPV-positive cancer cell lines.

Cell line	Origin	HPV subtype	Integration	E2 status	Copies
UM-SCC47	Lateral tongue	HPV16	Yes	Intact	15
CaSki	Cervix	HPV16	Yes	Intact	600
SiHa	Cervix	HPV16	Yes	Deleted	1–2

### Clinical specimens

The tumor tissues were collected from patients treated at the Department of Otorhinolaryngology, Head and Neck Surgery, University of the Ryukyus between December 2008 and May 2011. The ethics committee of the University of the Ryukyus specifically approved this study, and written informed consent was obtained from all patients. HPV16 infection was determined by PCR amplification and sequencing as reported previously [5,37]. Specimens from 10 representative OPSCC patients with HPV16 infection were investigated in this study (Table 2).

**Table 2:** Clinical characteristics of oropharyngeal cancer patients with HPV infection.

Patient NO.	HPV subtype	Sex	Age (y)	Tumor location	Histopathologic grade	TNM	Stage	Viral load^#^	E2/E6 rate
OPSCC-1	HPV16	M	60	Tonsil	Poorly differentiated	T3N2M1	ⅣC	359145	0.21
OPSCC-2	HPV16	M	41	Tonsil	Moderately differentiated	T2N2M0	ⅣA	1885523	0.14
OPSCC-3	HPV16	M	54	Tonsil	Poorly differentiated	T2N2M0	ⅣA	4083550	0.18
OPSCC-4	HPV16	F	70	Tonsil	Moderately differentiated	T2N2M0	ⅣA	4664217	0.12
OPSCC-5	HPV16	M	50	Tongue base	Well differentiated	T2N2M0	ⅣA	1819871	1.05
OPSCC-6	HPV16	M	60	Tongue base	Poorly differentiated	T2N2M0	ⅣA	1333430	0
OPSCC-7	HPV16	M	58	Tonsil	Poorly differentiated	T3N2M0	ⅣA	2115	0.35
OPSCC-8	HPV16	F	74	Tonsil	Moderately differentiated	T2N2M0	ⅣA	14204321	0.12
OPSCC-9	HPV16	M	67	Tonsil	Well differentiated	T2N1M0	Ⅲ	62634	0.02
OPSCC-10	HPV16	M	50	Tonsil	Poorly differentiated	T2N1M0	Ⅲ	237	0.7

OPSCC, oropharyngeal squamous cell carcinoma

^**#**^ E6 copies in 50 ng sample of DNA.

### Bisulfite modification, PCR amplification, TA cloning, and DNA sequencing

DNA extraction from cells and tissue samples was performed using a Gentra Purification Tissue Kit (QIAGEN, Valencia, CA) as reported previously [5], then DNA (1–2 μg) was bisulfite-converted using an EpiTect^®^ Plus Bisulfite Kit (QIAGEN, Valencia, CA) according to the manufacturer’s protocol.

The modified DNA was amplified in 3 amplicons with bisulfite-sequencing PCR (BSP) primer assays and termed BSP-1, BSP-2, and BSP-3 (S1 Table). For UM-SCC47 and some tissue samples that were negative on PCR amplification using the primer sets BSP-1 or BSP-2, we adopted the primer sets BSP-5 and BSP-6 instead (S1 Table). The BSP was carried out in a 25 μl volume containing 0.2 mM of each of the 4 dNTPs, 2 mM MgCl_2_, 10 pmol of each primer, 1.25 units AmpliTaq DNA Polymerase (Applied Biosystems, Carlsbad, CA), and 300 ng bisulfite-modified DNA. The BSP conditions were 95°C for 5 min, followed by 45 cycles at 95°C for 1 min, 54°C for 1 min, and 72°C for 2 min, with a final extension at 72°C for 7 min. As reported previously [5], PCR products were purified using a Wizard^®^ SV Gel and PCR Clean-Up System (Promega, Madison, WI) and then cloned into the pCR™4-TOPO^®^ vector (Invitrogen, Carlsbad, CA) for sequencing. For each amplicon, at least 6 individual clones were sequenced using an ABI PRISM 3130xl Genetic Analyzer (Applied Biosystems).

### Methylation-specific PCR

Nested PCR was carried out to analyze the overall methylation status of the LCR and methylation variation after demethylation treatment. The primers used for nested PCR are shown in S1 Table, and their products cover 7 methylated/unmethylated (M/U) CpG sites in the 5′-LCR and enhancer region. Bisulfite-modified DNA was first amplified by PCR with the BSP-6 primer set. The BSP was carried out in a 25 μl volume containing 0.2 mM of each of the 4 dNTPs, 2 mM MgCl_2_, 10 pmol of each primer, 1.25 units AmpliTaq DNA Polymerase (Applied Biosystems), and 100 ng bisulfite-modified DNA. The BSP conditions were 95°C for 5 min, followed by 25 cycles at 95°C for 60 s, 54°C for 60 s, and 72°C for 2 min, with a final extension at 72°C for 7 min. Then the product from the first round of PCR was subjected to a second round of amplification using methylation-specific PCR primer sets (S1 Table), so methylated and unmethylated sequences could be amplified separately. The PCR was carried out in a 20 μl volume containing 0.2 mM of each of the 4 dNTPs, 2 mM MgCl_2_, 10 pmol of each primer, 1.0 unit AmpliTaq DNA Polymerase (Applied Biosystems), and 3 μl product of the first round of BSP. The PCR conditions were 95°C for 5 min, followed by 25 cycles of 95°C for 40 s, 58°C for 40 s, and 72°C for 1 min, with a final extension at 72°C for 7 min.

### Knockdown of E6 and E7

Silencer^®^ Select siRNA targeting HPV16 E6 and E7 (5′-GUA UGG AAC AAC AUU AGA A-3′), Silencer^®^ siRNA targeting GAPDH (positive control), and scrambled siRNA (negative control) were obtained from Ambion (Life Technologies Japan, Tokyo, Japan). For siRNA transfection, the cells were seeded into 6-well plates (1.0 × 10^5^ cells/well). After overnight recovery, the cells were transfected individually with 60 nM siRNA using a Lipofectamine RNAiMax^®^ Reagent (Invitrogen) according to the manufacturer’s protocol. The cells were subjected to total RNA extraction at 72 h after transfection, and growth inhibition, apoptosis, and cell cycle arrest were analyzed at 96 h after transfection.

### Detection of proliferation and apoptosis, and cell cycle analysis

To detect growth inhibition after demethylation treatment, the cells were seeded in triplicate into 96-well plates (1,500 cells/well). After overnight recovery, the cells were treated continuously with freshly prepared 5-aza-dc from day 1 to day 7. Cell proliferation at different time points was measured using a CellTiter 96^®^ Aqueous One Solution Cell Proliferation Assay (MTS; Promega, Madison, WI) and absorbance (OD 490 nm) was measured using a microreader (SH-1000; Wakenyaku, Kyoto, Japan). After knockdown of E6 and E7, we mainly focused on inhibition of proliferation at 96 h. Thus, we utilized trypan blue exclusion for cell growth inhibition after siRNA. The cells were seeded in triplicate into 6-well plates (1.0 × 10^5^ cells/well). At 96 h after transfection of siRNAs, the cells were detached and stained with 0.4% trypan blue solution, and the growth inhibition rate could be determined by counting and comparing dye-exclusive cells between the groups transfected with scrambled siRNA or siRNA targeting E6 or E7. Moreover, the dead cells after loss of E6 and E7 could be compared between different groups.

To assess the rate of apoptosis, annexin-V and 7-AAD staining (Guava Nexin Reagent; Millipore, Hayward, CA) was examined by flow cytometry (Guava EasyCyte Plus Flow Cytometer; Millipore). The cells were seeded into 6-well plates (1.0 × 10^5^ cells/well). After overnight recovery, the cells were treated with 5-aza-dc or transfected with siRNA as described above. According to the manufacturer’s instructions, the log of annexin-V-PE and 7-AAD was displayed on the x- and y-axes of the cytogram, respectively. Annexin-V-positive cells plotted in the lower right and upper right quadrants were evaluated as early apoptotic cells and late apoptotic/dead cells, respectively.

Cell cycle analysis was carried out using propidium iodide staining to determine DNA content. The cells were treated with 5-aza-dc or transfected with siRNA as described above. After collecting the culture medium, the cells were washed twice with phosphate-buffered saline (PBS) and detached. The detached and floating cells in the culture medium and PBS were collected by centrifugation. After discarding the supernatant and fixing the cells with ice-cold ethanol, the cells were stained with the Guava cell cycle reagent and data were acquired using a Guava EasyCyte Plus Flow Cytometer. Modfit LT™ 4.0 software (Verity Software House, Topsham, ME) was used to analyze the cell cycle results.

### Quantitative real-time PCR

To detect E6 and E7 expression after 5-aza-dc treatment or E6 and E7 knockdown, total RNA was extracted from the cells and converted to cDNA as reported previously [36]. β-Actin was adopted as an internal control; β-actin, E6, and E7 were amplified using the ABI Prism 7300 Sequence Detection System (Applied Biosystems) and TaqMan^®^ PCR Master Mix II (Roche Molecular Systems, Foster City, CA) as described previously [32,36]. The relative expressions of E6 and E7 were calculated as E6/β-actin and E7/β-actin.

### Immunocytochemistry

The cells were seeded into 6-well plates with sterilized coverslips at the bottom of the well and treated with 5-aza-dc as described above. After the cells were fixed with 4% paraformaldehyde for 20 min at room temperature, the coverslips were blocked with 3% H_2_O_2_ for 5 min at room temperature. Then, the coverslips were subjected to overnight incubation at 4°C with a primary anti-HPV16-E6 antibody (C1P5, 1:100; Santa Cruz Biotechnology, Dallas, TX) or anti-HPV16-E7 antibody (ED17, 1:100; Santa Cruz Biotechnology). The cells were washed with PBS and incubated with a horseradish peroxidase-conjugated goat anti-mouse secondary antibody (MTM Laboratories AG, Heidelberg, Germany) for 30 min at room temperature. The sections were then color-developed in 3–3′-diaminobenzidine for 3 min and counterstained with hematoxylin.

### Statistical analysis

Comparisons of two groups were performed using Student’s t-test or non-parametric tests. The difference in frequencies between two groups was analyzed using the Pearson’s χ^2^ test or Fisher’s exact test. The associations between methylation status and viral load, and E2/E6 rate were analyzed by Spearman’s rank correlation. A P value < 0.05 was considered statistically significant. All statistical tests were two-sided, and all analyses were performed with SPSS software v12.0 (SPSS, Inc., Chicago, IL).

## Results

### Different LCR methylation patterns in HPV16-positive cancer cell lines

**Fig 1:**
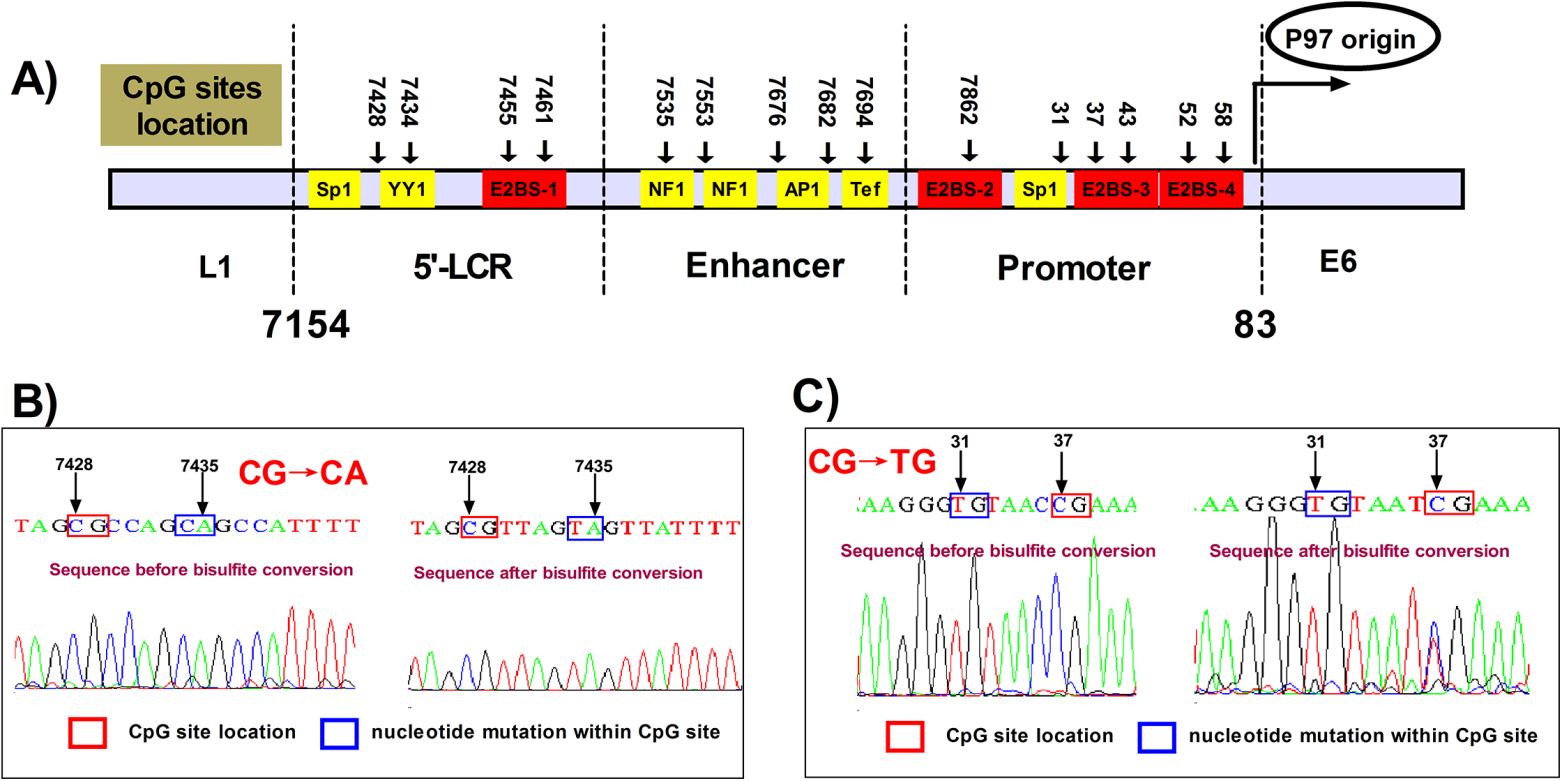
Structure of HPV16 LCR and location of the CpG sites. **A)** The structure of HPV16 LCR and location of the CpG sites. Transcription factor binding sites are also shown. **B, C)** Nucleotide mutations at nt7435 (G→A) and nt31 (C→T) of UM-SCC47 cells affected the presence of the CpG sites.

**Fig 2:**
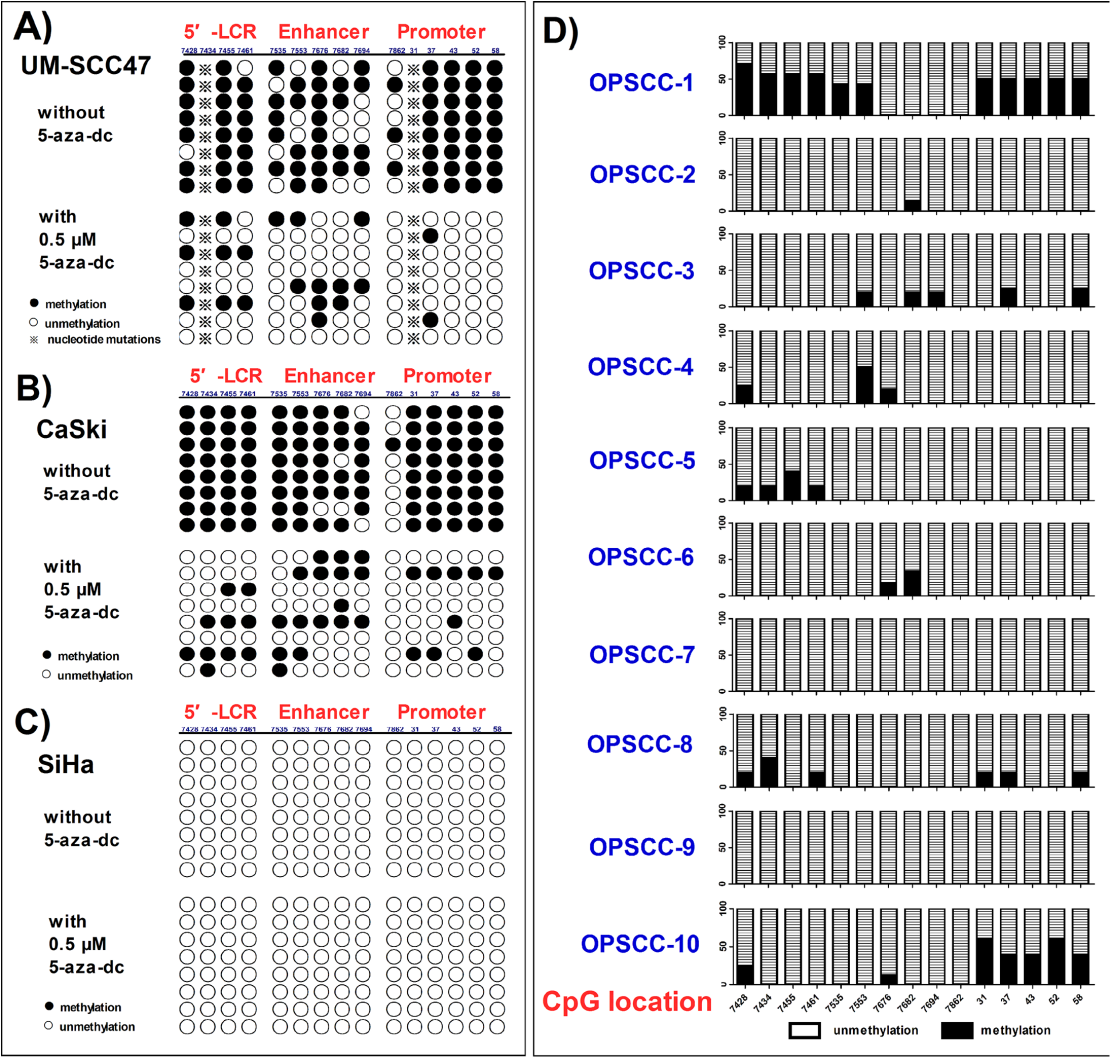
Methylation of CpG sites within the LCR in HPV-positive cancer cells before and after demethylation and in tissues. Eight individual clones were sequenced to identify the presence and change of each methylated CpG dinucleotide before and after demethylation induced by 0.5 μM 5-aza-dc for 96 h in **A)** UM-SCC47, **B)** CaSki, and **C)** SiHa cells. Filled circles represent methylated CpGs (meCpGs), open circles represent unmethylated CpGs, and asterisks represent the absence of the CpG sites. **D)** Methylation of CpG sites in tissues. For every CpG site, 6 clones were sequenced to identify the presence and frequency of meCpG clones. The percentages of meCpG clones are indicated by the left vertical bar. At the bottom of the figure, the positions of the CpG sites are marked.

A total of 15 CpG sites located within the LCR of the HPV16 genome, which is rich in regulatory elements, were investigated in this study (Fig 1A and S1 Fig). As this was the first examination of the LCR of UM-SCC47 cells, we acquired the LCR sequence by PCR using 3 primer sets, that is, LCR-1, -2, and -3, as shown in S1 Table. Since 2 nucleotide (nt) mutations (nt7435 and nt31) altered the presence of the CpG sites (Fig 1B and 1C, and S1 Fig), only 13 CpG sites were assessed in UM-SCC47 cells (S1 Fig). In this study, UM-SCC47 and CaSki cells showed hypermethylation within the LCR (79.8% and 90.0%, respectively) (Fig 2A and 2B). The CpG site (nt7862) located within E2BS-2 was relatively hypomethylated (37.5% in UM-SCC47 and 12.5% in CaSki), whereas the CpG sites within the E2BSs other than nt7862 were hypermethylated in UM-SCC47 (97.9%) and CaSki (100%) cells. By contrast, all CpG sites within the LCR in SiHa cells were unmethylated (0/120) (Fig 2C). Thus, SiHa cells could be used as a negative control for further investigation of demethylation of meCpG sites.

### 5-Aza-dc induces CpG hypomethylation within the LCR in UM-SCC47 and CaSki cells

As a strong demethylation reagent, 5-aza-dc has shown therapeutic effects on hematologic malignant diseases and some solid tumors, including HNSCC [38]. In the present study, treatment with 0.5 μM 5-aza-dc for 96 h showed optimal demethylation potency (0.1–5 μM tested) (S2 Fig).

After 0.5 μM 5-aza-dc treatment, the rates of meCpGs within the LCR in UM-SCC47 and CaSki cells were significantly decreased to 19.2% (20/104; P < 0.001) and 29.2% (35/120; P < 0.001), respectively, while no change was found in SiHa cells (0%, 0/120) (Fig 2). Interestingly, in UM-SCC47 cells treated with 5-aza-dc, not only were the meCpGs in the promoter region demethylated significantly more than in the 5′-LCR and enhancer region (P = 0.038), but also meCpGs within E2BSs had significantly greater demethylation variation (P = 0.025).

### LCR demethylation decreases E6 and E7 expression in UM-SCC47 and CaSki cells

**Fig 3:**
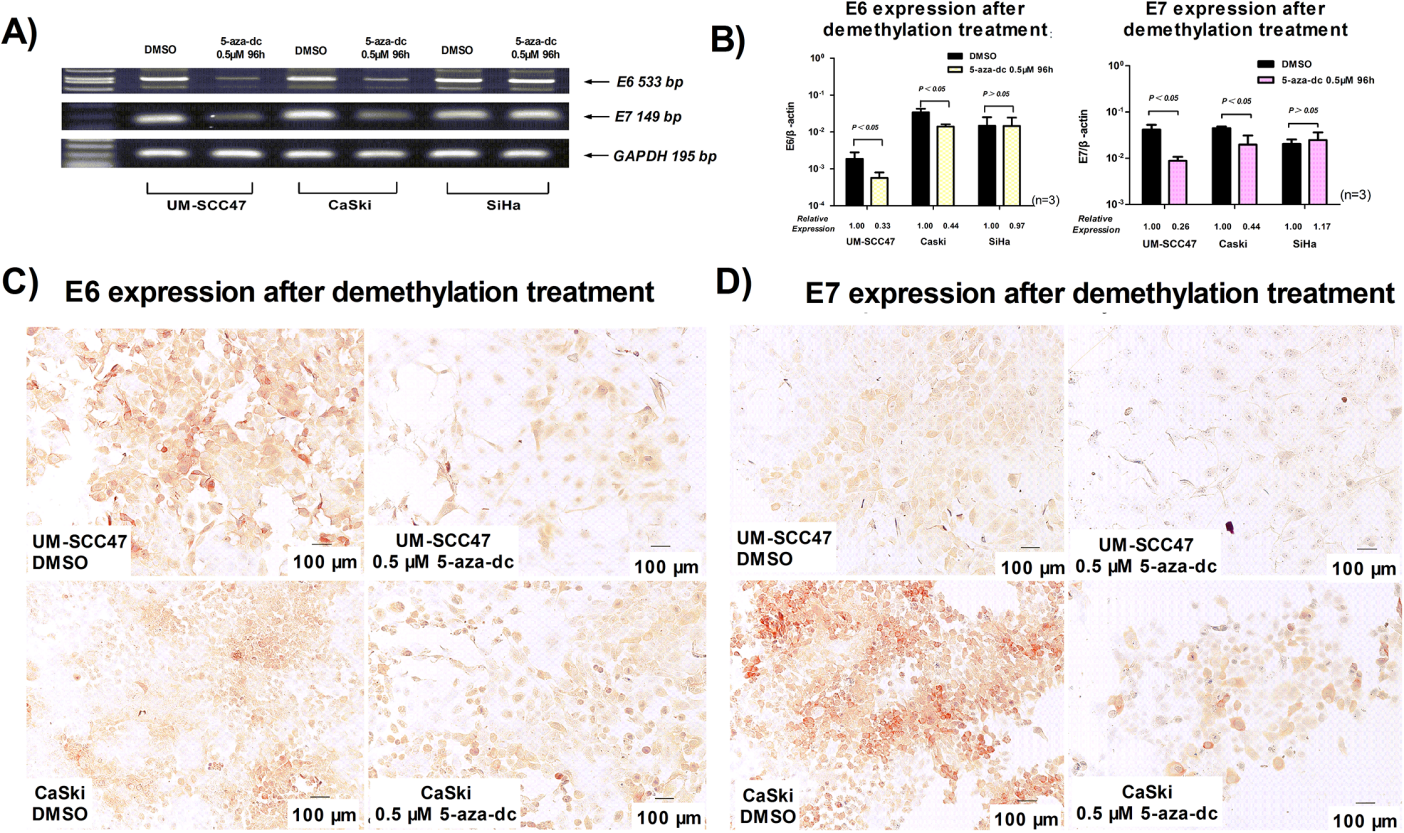
Demethylation of HPV16 LCR CpG sites decreases E6 and E7 expression. Demethylation of the CpG sites induced by 0.5 μM 5-aza-dc for 96 h decreased E6 and E7 expression, which was detected by reverse transcription PCR **A)** and quantitative real-time PCR **B)**; E6 and E7 were clearly decreased in UM-SCC47 and CaSki cells, but there was no obvious change in SiHa cells. The values (mean ± SD) were obtained from at least 3 independent experiments. **C)** E6 and **D)** E7 protein after demethylation of the CpG sites was detected by immunocytochemistry; E6 and E7 oncoproteins were distributed in the cytoplasm and nucleus of UM-SCC47 and CaSki cells with a strong staining intensity (×100, bar = 100 μm), and both the percentages and intensity of stained cells were clearly decreased after demethylation (×100, bar = 100 μm).

Upon demethylation treatment with 0.5 μM 5-aza-dc for 96 h, the expression of E6 and E7 mRNA was significantly decreased in UM-SCC47 (P = 0.021 and P = 0.008, respectively) and CaSki cells (P = 0.017 and P = 0.023, respectively), but not in SiHa cells (Fig 3A and 3B). Furthermore, we detected E6 and E7 proteins in UM-SCC47 and CaSki cells by immunocytochemistry. E6 and E7 proteins were localized in the cytosol and nucleus of UM-SCC47 and CaSki cells, and the percentage of stained cells and the staining intensity were significantly decreased after demethylation (Fig 3C and 3D).

### Hypomethylation of HPV16 LCR reduces cell proliferation, increases apoptosis, and induces cell cycle arrest in UM-SCC47 and CaSki cells

**Fig 4:**
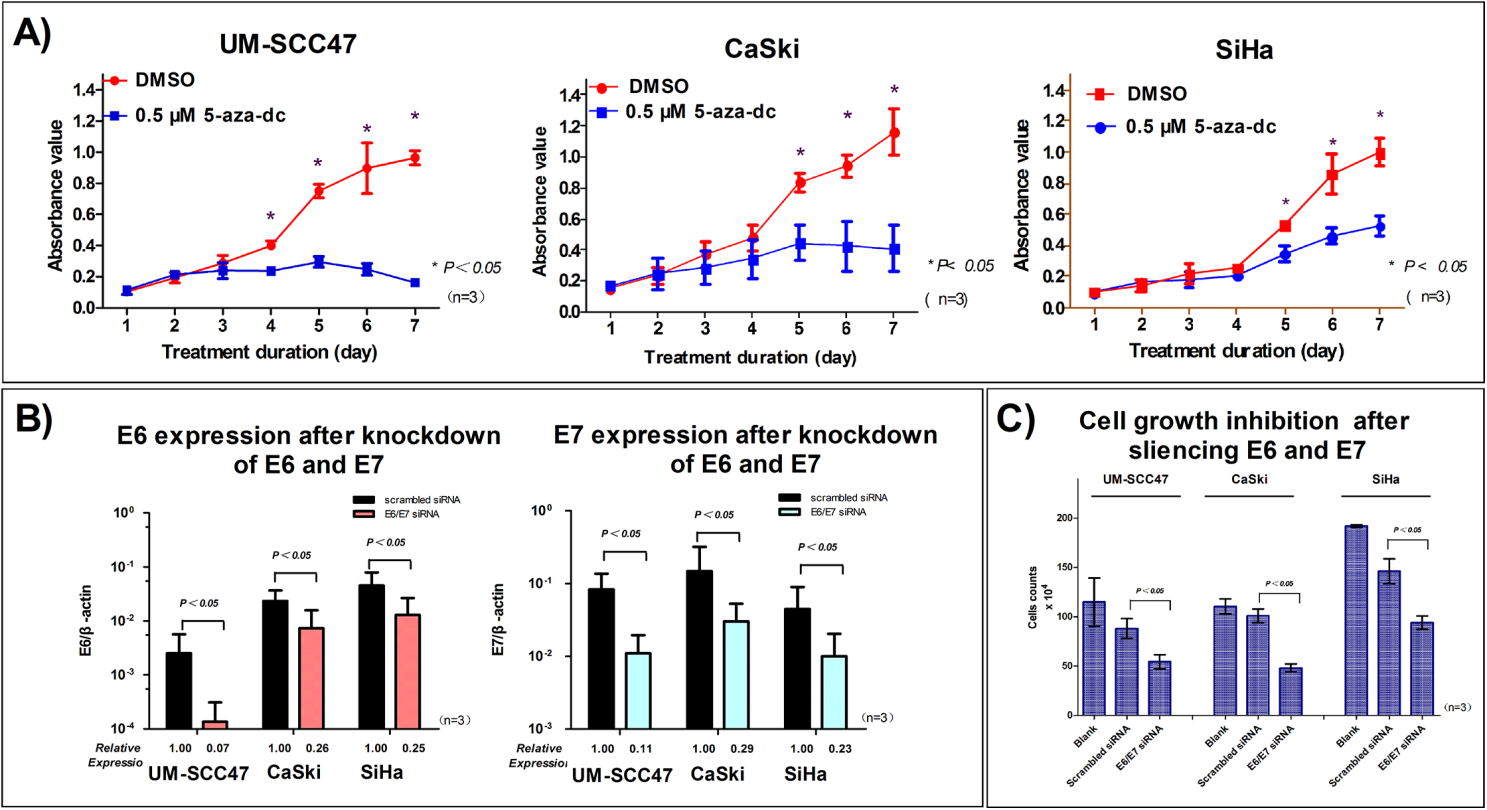
Demethylation of HPV16 LCR CpG sites and knockdown of E6 and E7 inhibit cell growth in HPV-positive cancer cells. **A)** After demethylation of the CpG sites, cell growth was strongly inhibited in UM-SCC47 and CaSki cells, but not in SiHa cells. **B)** E6 and E7 expression was significantly decreased after siRNA transfection. **C)** Cell growth was inhibited at 96 h after E6 and E7 knockdown. All data are means ± SEM from 3 independent experiments in **A)** and **C)**, and means ± SD from 3 independent experiments in **B)**.

To examine whether demethylation of HPV16 LCR by 5-aza-dc affects cell bioactivity, we analyzed proliferation, apoptosis, and cell cycle arrest. The growth and physical properties of all 3 cell lines were not obviously changed in the 2 days after 5-aza-dc treatment. However, 4 days after treatment, the growth of UM-SCC47 and CaSki cells was almost completely inhibited (Fig 4A), which was in accordance with the optimum treatment duration and the subsequent loss of E6 and E7 expression. Growth inhibition of SiHa cells resulting from 5-aza-dc treatment was also observed, which may be associated with cytotoxicity of 5-aza-dc induced by the accumulated incorporation into cellular DNA [39]; however, the effect was modest compared with CaSki and UM-SCC47 cells (Fig 4A).

**Fig 5:**
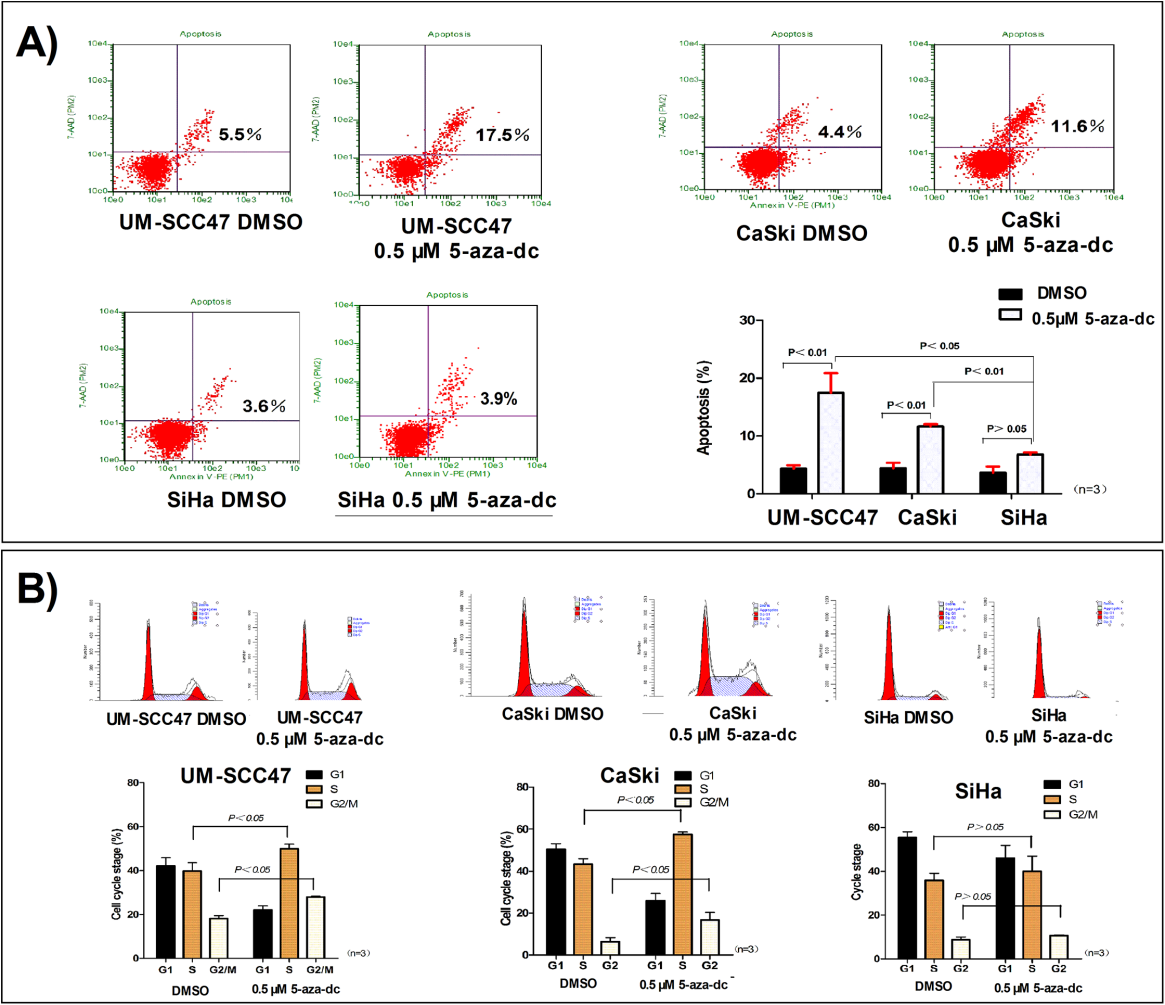
Demethylation of HPV16 LCR CpG sites induces apoptosis and cell cycle arrest. **A)** After demethylation of the CpG sites, the number of apoptotic cells increased significantly in UM-SCC47 and CaSki cells, but not in SiHa cells. **B)** After demethylation of the CpG sites, the number of cells arrested during both S and G2/M phases increased in UM-SCC47 and CaSki cells, but not in SiHa cells. All data are means ± SEM from 3 independent experiments.

Apoptotic cells were quantified by annexin-V-PE staining and flow cytometry. With demethylation of meCpGs by 5-aza-dc treatment, the apoptotic rates of UM-SCC47 and CaSki cells were significantly increased (P = 0.026 and P = 0.001, respectively). Moreover, 5-aza-dc increased the number of UM-SCC47 and CaSki cells arrested during S phase (P = 0.048 and P = 0.003, respectively) and G2/M phase (P = 0.002 and P = 0.044, respectively) (Fig 5). By contrast, in SiHa cells, which have a single unmethylated HPV genome, no significant changes in apoptosis or cell cycle arrest at S and G2/M phases were observed (Fig 5).

### Cell bioactivity changes after demethylation treatment are associated with the loss of E6 and E7 expression

**Fig 6:**
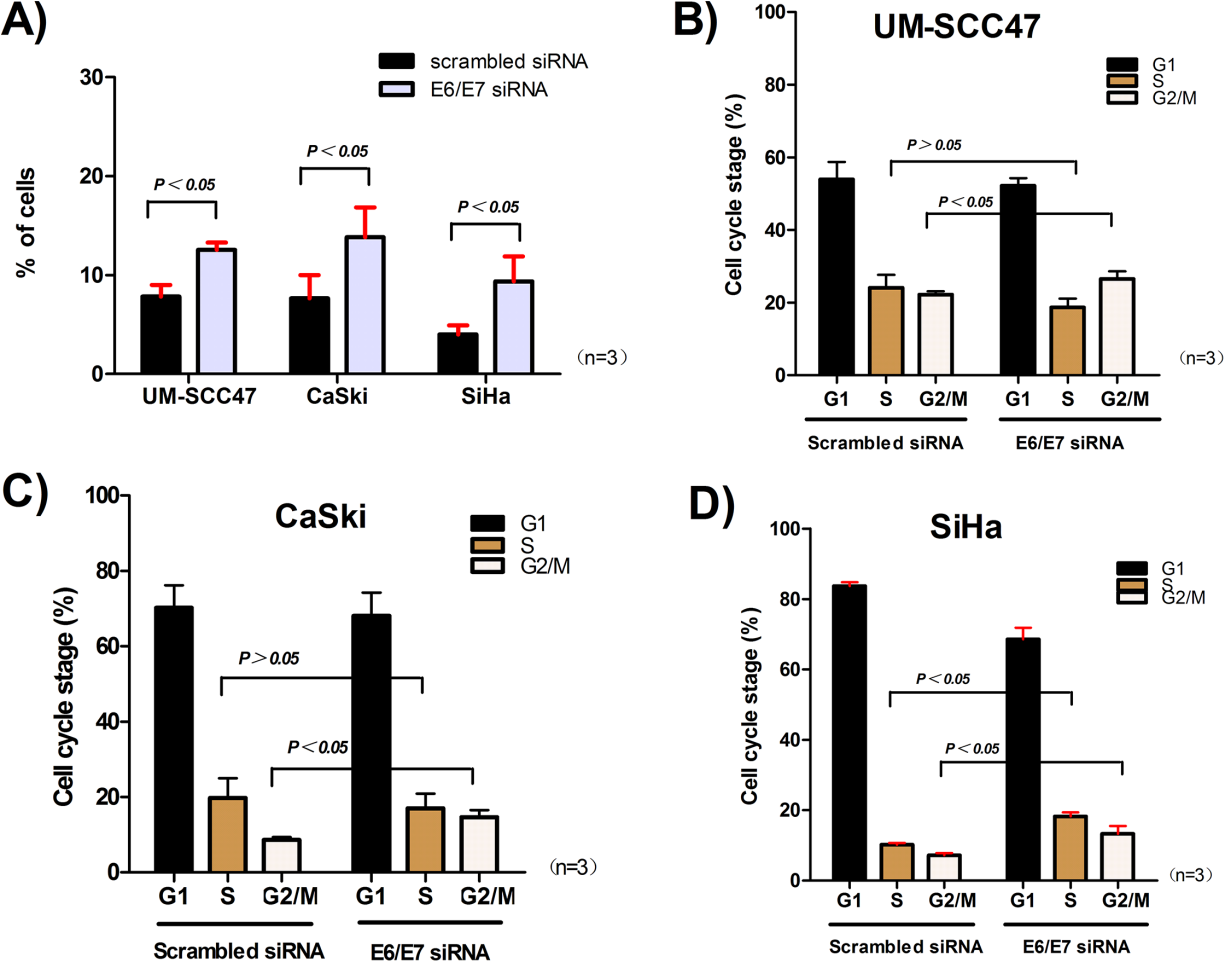
Apoptosis and cell cycle arrest after E6 and E7 knockdown. **A)** The number of apoptotic cells increased at 96 h after E6 and E7 knockdown in UM-SCC47, CaSki, and SiHa cells. The cells were arrested in G2/M phase in **B)** UM-SCC47 and **C)** CaSki cells, and in both G2/M and S phases in **D)** SiHa cells. All data are means ± SEM from 3 independent experiments.

In order to determine whether the bioactivity changes following 5-aza-dc treatment were associated with the loss of E6 and E7 expression caused by demethylation, we transfected the 3 cell lines with siRNA to knock down E6 and E7 (Fig 4B). At 96 h after transfection, the growth of UM-SCC47, CaSki, and SiHa cells was inhibited (P = 0.044, P = 0.006, and P = 0.022, respectively) (Fig 4C); moreover, apoptosis (P = 0.026, P = 0.050, and P = 0.016, respectively) and G2/M phase arrest (P = 0.027, P = 0.035, and P = 0.012, respectively) were induced (Fig 6). These finding were in accordance with the effects on cells after demethylation. Our results suggest that the significant bioactivity changes after 5-aza-dc treatment might be induced by decreased E6 and E7 expression caused by meCpG demethylation. However, it should be noted that the dramatic growth inhibition after transfection suggested the E6/E7 siRNA possibly had off-target effects.

### Different methylation patterns in HPV-positive OPSCC patients

We collected specimens of 10 OPSCC patients with HPV16 infection for analysis of their LCR methylation patterns (Table 2). The mean methylation rate in the 10 specimens was 9.5% for the entire LCR region, 13.9% in the 5′-LCR, 6.0% in the E6 enhancer, and 9.5% in the p97 promoter (Fig 2D). More than 70% of the CpG sites (111/150) were completely unmethylated, while 26.0% (39/150) were heterogeneously methylated (≥ 1 meCpG clone). All CpG clones were unmethylated at nt7862, which is in line with the clear hypomethylation status in UM-SCC47 and CaSki cells. However, at the other CpG sites, at least 1 meCpG clone was detected.

Although the sample size was relatively small, we performed further analysis and classified the methylation profiles as hypermethylation and hypomethylation. Two patients with tonsillar cancer (OPSCC-1 and OPSCC-10) had a hypermethylation status (41.6% and 40.0%, respectively) in the p97 promoter. However, in the other patients, only sporadic meCpG clones or all unmethylated CpG clones were detected, and the mean methylation rate was 4.1% (24/585). The distinct methylation profiles in the OPSCC patients were very similar to the methylation types in the HPV-positive cancer cell lines. Furthermore, we analyzed the relationships between methylation status and viral load (E6 copies), and E2/E6 rate [36]. After excluding 2 cases (OPSCC-5 and OPSCC-7) with outliers in the scatter plot, the methylation frequency of the p97 promoter was positively correlated with E2/E6 rate (r = 0.759, P = 0.029). However, no significant association was found between the methylation frequency of the p97 promoter and viral load (P = 0.220).

## Discussion

Previous studies of HPV16 LCR methylation were mainly based on cervical cells and clinical samples. Upon recombination with the cellular genome, methylation status of the HPV16 genome may be altered and dependent on the type of integration event and probably viral load [30]. In the present study, HPV16 LCR was hypermethylated in cervical CaSki cells with tandemly integrated HPV16 genome (a type 2 integrant) and unmethylated in SiHa cells with integration of individual HPV16 genomes (a type 1 integrant). These findings were in line with the results of previous studies [7,15,25,32]. We first identified HNSCC UM-SCC47 cells containing an intact E2 gene of the HPV16 genome and a hypermethylated LCR as cervical CaSki cells, in which HPV genomes are integrated into the cellular genome in tandem arrays [20]. The methylation of E2BSs in HPV16 LCR was correlated with the expression level of the oncogenic E6 and E7, and demethylation of HPV16 LCR in UM-SCC47 cells repressed E6 and E7 expression, inhibited proliferation, and ultimately caused cell cycle arrest at G2/M. Our findings suggest that methylation within HPV16 LCR also plays a vital role in the viral life cycle in HNSCC cells containing a type 2 integrant HPV16 genome. Furthermore, methylation can also be interpreted as an alternative defense mechanism to silence viral replication and transcription [40], which may represent a novel mechanism by which the HPV genome is converted from a productive infectious agent to a novel carcinogen.

It has been shown that completely methylated HPV16 genome is transcriptionally inactive [41] and previous studies suggested that heavy methylation is not needed for LCR E2BSs to influence p97 promoter activity [27,42]. Repression of the promoter can restore the normal functions of p53 and pRb, as well as other targets of E6 and E7 [43–45]. Moreover, reintroduction of E2 in association with either E6 or E7 under the control of an E2-independent promoter has been used to clarify further the functions of E6 and E7 in transformed cells [43,45]. Our data showed the CpG sites in the 5′-LCR and promoter region of UM-SCC47 cells, including all E2BSs, were methylated preferentially, and 5-aza-dc demethylation was more effective for the meCpGs in the promoter region and E2BSs than the other CpG sites within HPV16 LCR in UM-SCC47 cells, suggesting that these crucial sites and region may be vulnerable to methylation in carcinogenesis and demethylation. However, the CpG at nt7862, which is located in the linker region between the enhancer and promoter, tended to be hypomethylated in both UM-SCC47 and CaSki cells, as well as OPSCC specimens, with similar results also reported previously [24,25,29,30,33]. This suggests that the CpG site may be involved in the viral origin of replication and its unmethylation status is often retained to maintain the viral life cycle. Further studies are necessary to explain this phenomenon.

Studies have demonstrated that HPV-positive HNSCCs have a better response to radiotherapy and/or chemotherapy than HPV-negative HNSCCs [46,47]. However, there is a subset of HPV-positive HNSCCs that is associated with a poor prognosis [48,49], which may be related to epigenetic modification of the viral and host genomes, as epigenetic changes are reversible. Our results indicated that methylation status of the LCR region was closely related to E6 and E7 expression and was critical in maintaining the bioactivity of cervical epithelial and HNSCC cells. For HPV-positive cancer cells with a hypermethylated LCR, meCpG demethylation and thus reactivation of the E2-mediated repression of E6 and E7 oncoprotein expression might be a novel target for a certain subgroup of HPV-positive HNSCC patients. Our results showed that a 5-aza-dc concentration of 0.5 μM produced an optimal demethylating effect, which was in line with the inherent characteristics of the drug [50], that is, effective demethylation with low cytotoxicity at a low dose but decreased demethylation ability at a high dose. Moreover, although the E6/E7 siRNA had likely some off-target effects, the consistency between the demethylation effect duration and cell responses, and the changes in cytoactivity after knockdown of E6 and E7 further supported our findings, whereas the S-phase arrest of the cells was most likely related to the inherent characteristics of 5-aza-dc [51]. Recently, we reported the potential use of star-shaped copolymers to co-deliver docetaxel and MMP-9 siRNA plasmids in cancer therapy [52]; thus, demethylation drugs in combination with such copolymers may increase the specificity of targeted therapy in the future.

Balderas-Loaeza et al [33] previously reported hypermethylation of HPV16 LCR in 10 out of 12 HPV16-positive oral carcinomas; however, OPSCC has a higher HPV infection rate among HNSCC, and a subgroup of OPSCC has causally been linked to HPV16 infection. Our *in vitro* findings verified the importance of HPV16 LCR methylation in OPSCC; most OPSCC clinical specimens showed a relative hypomethylation profile of the LCR, and the mean methylation rate of 9.5% was far below the percentage of 53.3% observed in cervical carcinoma [24]. Of interest, 2 different methylation patterns of the LCR in HNSCC samples might be classified based on the methylation frequency of p97 promoter, that is, hypermethylation (≥40% as in UM-SCC47 and CaSki cells) and hypomethylation (<10% as in SiHa cells). Our findings are similar to those of Park et al in which 95.5% (21/22) of advanced-stage HNSCCs were unmethylated at ≥50% of the CpG sites within HPV16 LCR [32]. However, direct sequencing, and not TA cloning with sequencing, was used to evaluate methylation frequency in their study, which could introduce deviation when sequencing. In a more recent report, a mean methylation frequency of <10% was found in 81% of p97 promoters of HPV16-positive OPSCC patients by pyrosequencing [7], which is similar to our findings although no further analysis was conducted on the effects of methylation status.

In HPV-driven cancers, recombination between HPV and chromosomal DNA is frequent and likely necessary for carcinogenesis. In the present study, these 3 HPV16-positive cancer cell lines were established as two different integrant types with different methylation statuses, whereas the methylation frequency of p97 promoter varied and was correlated with the E2/E6 rate in OPSCC tissues, possibly suggesting the presence of mixtures of type 1 and type 2 integrant in different proportions. It should be noted that an unmethylation status with high E2/E6 rate was found in 2 OPSCC specimens, and we speculate that the methylation status might be lost along with a change of physical status from episome to integration (type 1 integrants) and cellular differentiation. Further research is needed to clarify the exact underlying mechanism. In a previous study by Kalantari et al, episomal HPV16 DNA replicating in undifferentiated cervical epithelial cells was moderately methylated throughout the enhancer-promoter segment and cellular differentiation led to a loss of methylation [30].

In summary, to our knowledge, this is the first study to map the methylation status of the LCR in HPV-positive HNSCC cells and OPSCC patients by BSP with TA cloning. Our study revealed two different methylation types of the LCR in HPV16-positive cancer cells and OPSCC patients, which may represent different carcinogenesis mechanisms of HPV-positive cancers. In addition, our findings highlighted the significance of the p97 promoter and E2BSs within the LCR on E2-mediated regulation of E6 and E7 oncoprotein expression in HPV16-positive HNSCC cells containing an intact E2 gene. Demethylation of the meCpGs in HPV16 LCR might be a potential target for a subgroup of HPV16-positive HNSCC patients, although hypermethylation of LCR p97 promoter was only found in a small subset of OPSCC patients. Further *in vitro* and *in vivo* experiments and clinical studies with large sample sizes are necessary to extend our results.

## Supporting Information

S1 Fig Sequence of the long control region (LCR) and the location of CpG sites in UM-SCC47 cells. Compared to the reference sequence (nt7291-104, Genebank: AF402678, S1A), 2 nucleotide mutations at 7435 and 31 altered the existence of CpG sites (highlighted in yellow, S1B), thus the sequence of HPV16 LCR in UM-SCC47 cells contains 13 CpG sites.
(TIF)

S2 Fig The demethylation efficiency of various concentrations and time courses of 5-aza-dc treatment in HPV16-positive cancer cells. The methylation status of LCR after various concentrations of 5-aza-dc and treatment durations was examined by MSP amplification using 2 primers sets (Met-MSP and UnM-MSP) (S1 Table), which can amplify the methylated **(M)** (256 bp) and unmethylated sequences **(U)** (256 bp) covering the 5’-LCR and enhancer, respectively. The same samples were equally loaded for Met-MSP and UnM-MSP amplification. A concentration of 0.5 μM and treatment duration of 96 h demonstrated strong potency for demethylation in UM-SCC47 and CaSki cells. The Met-MSP amplification for SiHa cells was always negative. **A)** Treatment with various concentrations (0.1–5 μM) for 96 h; **B)** treatment with 0.5 μM 5-aza-dc for various durations.
(TIF)

S1 Table Primers for PCR, bisulfite-sequencing PCR, and methylation-specific PCR of the long control region. (DOC)

S2 Table Primers of RT-PCR and qRT-PCR for detection of HPV16 E6 and E7 mRNA. (DOC)
