# Supplementary figures and images for: Effects of Methylation Status of CpG Sites within the HPV16 Long Control Region on HPV16-Positive Head and Neck Cancer Cells
Source: PLoS One. 2015 Oct 28;10(10):e0141245. doi: 10.1371/journal.pone.0141245 (PMC4625038; doi:10.1371/journal.pone.0141245)

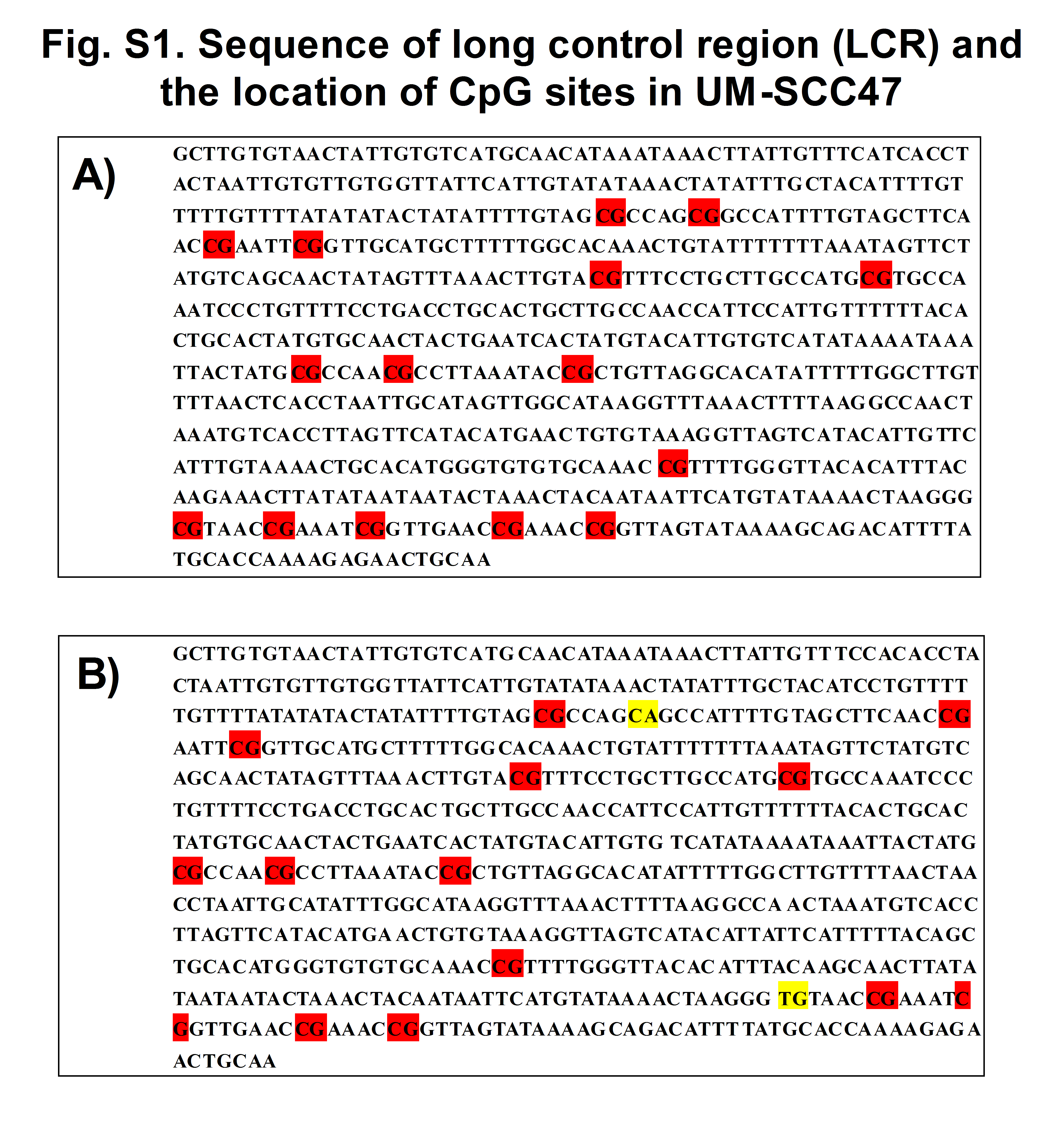

Supplement: S1 Fig — Compared to the reference sequence (nt7291-104, Genebank: AF402678, S1A), 2 nucleotide mutations at 7435 and 31 altered the existence of CpG sites (highlighted in yellow, S1B), thus the sequence of HPV16 LCR in UM-SCC47 cells contains 13 CpG sites. (TIF) [file pone.0141245.s001.tif]

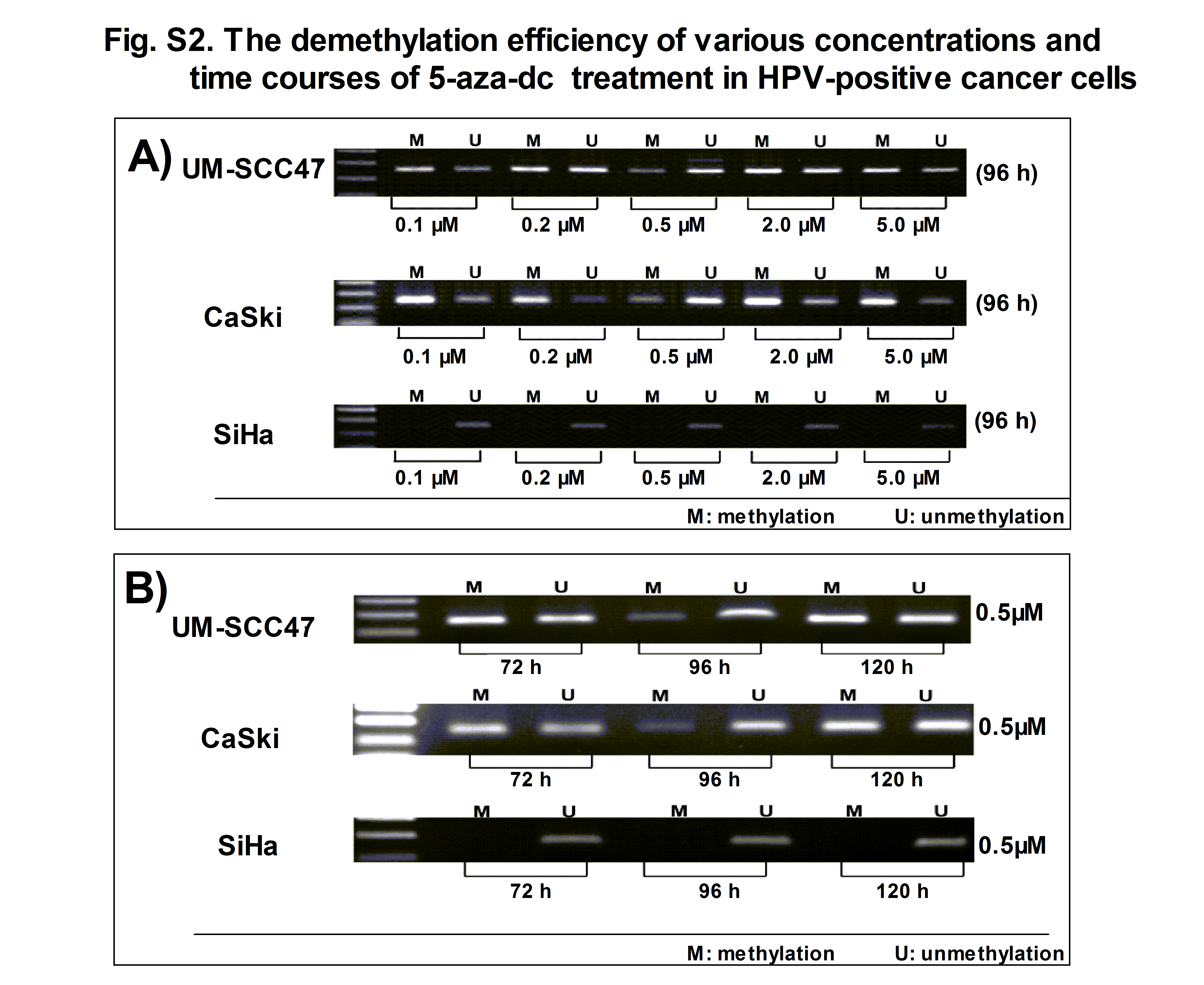

Supplement: S2 Fig — The methylation status of LCR after various concentrations of 5-aza-dc and treatment durations was examined by MSP amplification using 2 primers sets (Met-MSP and UnM-MSP) (S1 Table), which can amplify the methylated (M) (256 bp) and unmethylated sequences (U) (256 bp) covering the 5’-LCR and enhancer, respectively. The same samples were equally loaded for Met-MSP and UnM-MSP amplification. A concentration of 0.5 μM and treatment duration of 96 h demonstrated strong potency for demethylation in UM-SCC47 and CaSki cells. The Met-MSP amplification for SiHa cells was always negative. A) Treatment with various concentrations (0.1–5 μM) for 96 h; B) treatment with 0.5 μM 5-aza-dc for various durations. (TIF) [file pone.0141245.s002.tif]
