# Supplementary material for: Effects of Methylation Status of CpG Sites within the HPV16 Long Control Region on HPV16-Positive Head and Neck Cancer Cells
Source: PLoS One. 2015 Oct 28;10(10):e0141245. doi: 10.1371/journal.pone.0141245 (PMC4625038; doi:10.1371/journal.pone.0141245)
Supplement: S1 Table — (DOC) [file pone.0141245.s003.doc]

**Table S1. Primers for PCR, bisulfite-sequencing PCR and methylation-specific PCR of Long control region**

| **Amplicons** | | **Target** | **Primer** | **Position** | **Sequence (5’＞3’)** |
| --- | --- | --- | --- | --- | --- |
| **PCR** | |  |  |  |  |
|  | LCR-1 | 5’-LCR and | Forward | 7291--7314 | GCTTGTGTAACTATTGTGTCATG |
|  |  | enhancer | Reverse | 7584--7565 | GTGCAGGTCAGGAAAACAG |
|  | LCR-2 | enhancer and | Forward | 7528--7548 | ACTTGTACGTTTCCTGCTTG |
|  |  | promoter | Reverse | 7877--7856 | GTGTAACCCAAAATCGGTTTGC |
|  | LCR-3 | promoter | Forward | 7801--7780 | GTCACCCTAGTTCATACATGA |
|  |  |  | Reverse | 104--84 | TGCAGTTCTCTTTTGGTGC |
| **BSP** | |  |  |  |  |
|  | BSP-1 | 5’-LCR | Forward | 7271-7296 | GTGTATGTGTTTTTAAATGTTTGTGTCAC |
|  |  |  | Reverse | 7650-7622 | AATATACATAATAATTCAATAATTAC |
|  | BSP-2 | enhancer | Forward | 7622-7650 | GTAATTATTGAATTATTATGTATATTGTGC |
|  |  |  | Reverse | 7853-7829 | CAATATATAAAACATTAACACAT |
|  | BSP-3 | promoter | Forward | 7829-7853 | TTGTAAAATTGTATATGGGTGTGTG |
|  |  |  | Reverse | 161-135 | ACAACTCTATACATAACTATAATAACT |
|  | BSP-4 | 5’-LCR | Forward | 7298-7328 | ATTATTGTGTTATGTAATATAAATAAATTT |
|  |  |  | Reverse | 7575-7550 | AATCAAAAAAACAAAAATTTAACAC |
|  | BSP-5 | enhancer | Forward | 7505-7532 | TGTTAGTAATTATGGTTTAAATTTGTA |
|  |  |  | Reverse | 7788-7758 | AACTAAAATAACATTTAATTAACCTTAAAA |
|  | BSP-6 | 5’-LCR and | Forward | 7375-7405 | TAAATTATATTTGTTATATTTTGTTTTTGT |
|  |  | enhancer | Reverse | 7777-7747 | TAATTAACCTTAAAAATTTAAACCTTATAC |
| **MSP** | |  |  |  |  |
|  | Met-MSP | 5’-LCR and | Forward | 7438-7463 | TATTTTGTAGTTTTAATCGAATTCG |
|  |  | enhancer | Reverse | 7694-7670 | CGATATAAAACGTTAACGCATAAT |
|  | UnM-MSP | 5’-LCR and | Forward | 7438-7463 | TATTTTGTAGTTTTAATTGAATTTG |
|  |  | enhancer | Reverse | 7694-7670 | CAATATATAAAACATTAACACAT |

BSP, bisulfite-sequencing PCR; MSP, methylation-specific PCR; Met-MSP, methylation MSP; UnM-MSP, unmethylation MSP.
