# Supplementary material for: Effects of Methylation Status of CpG Sites within the HPV16 Long Control Region on HPV16-Positive Head and Neck Cancer Cells
Source: PLoS One. 2015 Oct 28;10(10):e0141245. doi: 10.1371/journal.pone.0141245 (PMC4625038; doi:10.1371/journal.pone.0141245)
Supplement: S2 Table — (DOC) [file pone.0141245.s004.doc]

**Table S2. Primers of RT-PCR and qRT-PCR for detection HPV16 E6 and E7 mRNA**

| Amplicons | Primer | Sequence (5’＞3’) | Length |
| --- | --- | --- | --- |
| RT-PCR | | | |
| HPV E6 | Forward | 5' – AATGTTTCAGGACCCACAGG – 3' | 533 bp |
|  | Reverse | 5' –TGCCCATTAACAGGTCTTCC – 3' |  |
| HPV E7 | Forward | 5' – TGAAATAGATGGTCCAGCTGG – 3' | 149 bp |
|  | Reverse | 5' – TGCCCATTAACAGGTCTTCC – 3' |  |
| GAPDH | Forward | 5' – CAAAGTTGTCATGGATGACC – 3' | 195 bp |
|  | Reverse | 5' – CCATGGAGAAGGCTGGGG – 3' |  |
| Real-time PCR | | |  |
| HPV E6 | Probe | FAM – CAGGAGCGACCCAGAAAGTTACCACAGTT – TAMRA | |
|  | Forward | 5' – GAGAACTGCAATGTTTCAGGACC – 3' | |
|  | Reverse | 5' – TGTATAGTTGTTTGCAGCTCTGTGC – 3' | |
| HPV E7 | Probe | FAM – CGCACAACCGAAGCGTAGAGTCACACT – TAMRA | |
|  | Forward | 5' – CCGGACAGAGCCCATTACAA – 3' | |
|  | Reverse | 5' – CGAATGTCTACGTGTGTGCTTTG – 3' | |
| β-actin | Probe | FAM – ATGCCCTCCCCCATGCCATCCTGCGT – TAMRA | |
|  | Forward | 5' – TCACCCACACTGTGCCCATCTACGA – 3' | |
|  | Reverse | 5' – CAGCGGAACCGCTCATTGCCAATGG – 3' | |
